# Effectiveness of Monovalent Rotavirus Vaccine Against Hospitalization With Acute Rotavirus Gastroenteritis in Kenyan Children

**DOI:** 10.1093/cid/ciz664

**Published:** 2019-07-20

**Authors:** Sammy Khagayi, Richard Omore, Grieven P Otieno, Billy Ogwel, John B Ochieng, Jane Juma, Evans Apondi, Godfrey Bigogo, Clayton Onyango, Mwanajuma Ngama, Regina Njeru, Betty E Owor, Mike J Mwanga, Yaw Addo, Collins Tabu, Anyangu Amwayi, Jason M Mwenda, Jacqueline E Tate, Umesh D Parashar, Robert F Breiman, D James Nokes, Jennifer R Verani

**Affiliations:** 1 Centre for Global Health Research, Kenya Medical Research Institute (KEMRI), Kisumu; 2 Centre for Geographic Medicine Research–Coast, KEMRI–Wellcome Trust Research Programme, Kilifi, and; 3 Division of Global Health Protection, Centers for Disease Control and Prevention (CDC)–Kenya, Kisumu, Kenya; 4 Emory Global Health Institute, Emory University, Atlanta, Georgia; 5 National Vaccines and Immunisations Programme, and; 6 Disease Surveillance and Response Unit, Ministry of Health, Nairobi, Kenya; 7 World Health Organization Regional Office for Africa, Brazzaville, Republic of Congo; 8 Viral Gastroenteritis Branch, Division of Viral Diseases, CDC, Atlanta, Georgia; 9 School of Life Sciences, and Zeeman Institute for Systems Biology and Infectious Disease Epidemiology Research, University of Warwick, Coventry, United Kingdom; 10 Division of Global Health Protection, CDC–Kenya, Nairobi, Kenya; and; 11 Division of Global Health Protection, CDC, Atlanta, Georgia

**Keywords:** rotavirus, acute gastroenteritis, vaccine effectiveness, Kenya

## Abstract

**Background:**

Rotavirus remains a leading cause of pediatric diarrheal illness and death worldwide. Data on rotavirus vaccine effectiveness in sub-Saharan Africa are limited. Kenya introduced monovalent rotavirus vaccine (RV1) in July 2014. We assessed RV1 effectiveness against rotavirus-associated hospitalization in Kenyan children.

**Methods:**

Between July 2014 and December 2017, we conducted surveillance for acute gastroenteritis (AGE) in 3 Kenyan hospitals. From children age-eligible for ≥1 RV1 dose, with stool tested for rotavirus and confirmed vaccination history we compared RV1 coverage among rotavirus positive (cases) vs rotavirus negative (controls) using multivariable logistic regression and calculated effectiveness based on adjusted odds ratio.

**Results:**

Among 677 eligible children, 110 (16%) were rotavirus positive. Vaccination data were available for 91 (83%) cases; 51 (56%) had 2 RV1 doses and 33 (36%) 0 doses. Among 567 controls, 418 (74%) had vaccination data; 308 (74%) had 2 doses and 69 (16%) 0 doses. Overall 2-dose effectiveness was 64% (95% confidence interval [CI], 35%–80%); effectiveness was 67% (95% CI, 30%–84%) for children aged <12 months and 72% (95% CI, 10%–91%) for children aged ≥12 months. Significant effectiveness was seen in children with normal weight for age, length/height for age and weight for length/height; however, no protection was found among underweight, stunted, or wasted children.

**Conclusions:**

RV1 in the Kenyan immunization program provides significant protection against rotavirus-associated hospitalization which persisted beyond infancy. Malnutrition appears to diminish vaccine effectiveness. Efforts to improve rotavirus uptake and nutritional status are important to maximize vaccine benefit.

(See the Major Article by Otieno, et al on pages 2306–13, and the Editorial Commentary by Steele and Groome on pages 2314–16.)

Rotavirus remains a leading cause of diarrheal illness and deaths among children worldwide. In 2013, rotavirus infection led to approximately 215 000 deaths among children aged <5 years, with more than half occurring in sub-Saharan Africa [1]. However, rotavirus acute gastroenteritis (AGE) is vaccine-preventable. Three live oral rotavirus vaccines are World Health Organization (WHO) prequalified and available: a monovalent strain (RV1; Rotarix, GlaxoSmithKline) and a pentavalent strain (RV5; RotaTeq, Merck Vaccines), were prequalified since 2006, while a monovalent vaccine, Rotavac (Bharat Biotech), was prequalified in early 2018. In 2009, WHO recommended that rotavirus vaccines be included in all national immunization programs, including low-resource settings in Africa and Asia [2, 3].

Although clinical trials of currently available rotavirus vaccines demonstrated high efficacy (generally >85%) against severe rotavirus disease in high-income settings, trials performed in resource-poor settings have found substantially lower efficacies (40%–60%) [4–6]. Given the high burden of severe rotavirus disease in low- and middle-income countries, as well as lower vaccine efficacy, it is important to monitor rotavirus vaccine effectiveness postintroduction in such settings. Evidence that rotavirus vaccines prevent rotavirus morbidity and mortality is accumulating from observational studies in African countries [7]. However, some studies were hampered by limited statistical power, and key questions such as duration of protection and effectiveness among malnourished children have not been satisfactorily addressed.

Kenya introduced RV1 (doses at 6 and 10 weeks) into routine immunization in July 2014. Early postintroduction data in Kenya have shown a reduction in the prevalence of rotavirus infection among children hospitalized with AGE [8, 9]. We evaluated RV1 effectiveness against rotavirus AGE hospitalization among Kenyan children.

## METHODS

The Rotavirus Immunization Program Evaluation in Kenya (RIPEK) was established as a collaboration among institutions with well-established rotavirus surveillance to provide country-wide data. This study examined vaccine effectiveness using a case-control design.

**Figure 1.**
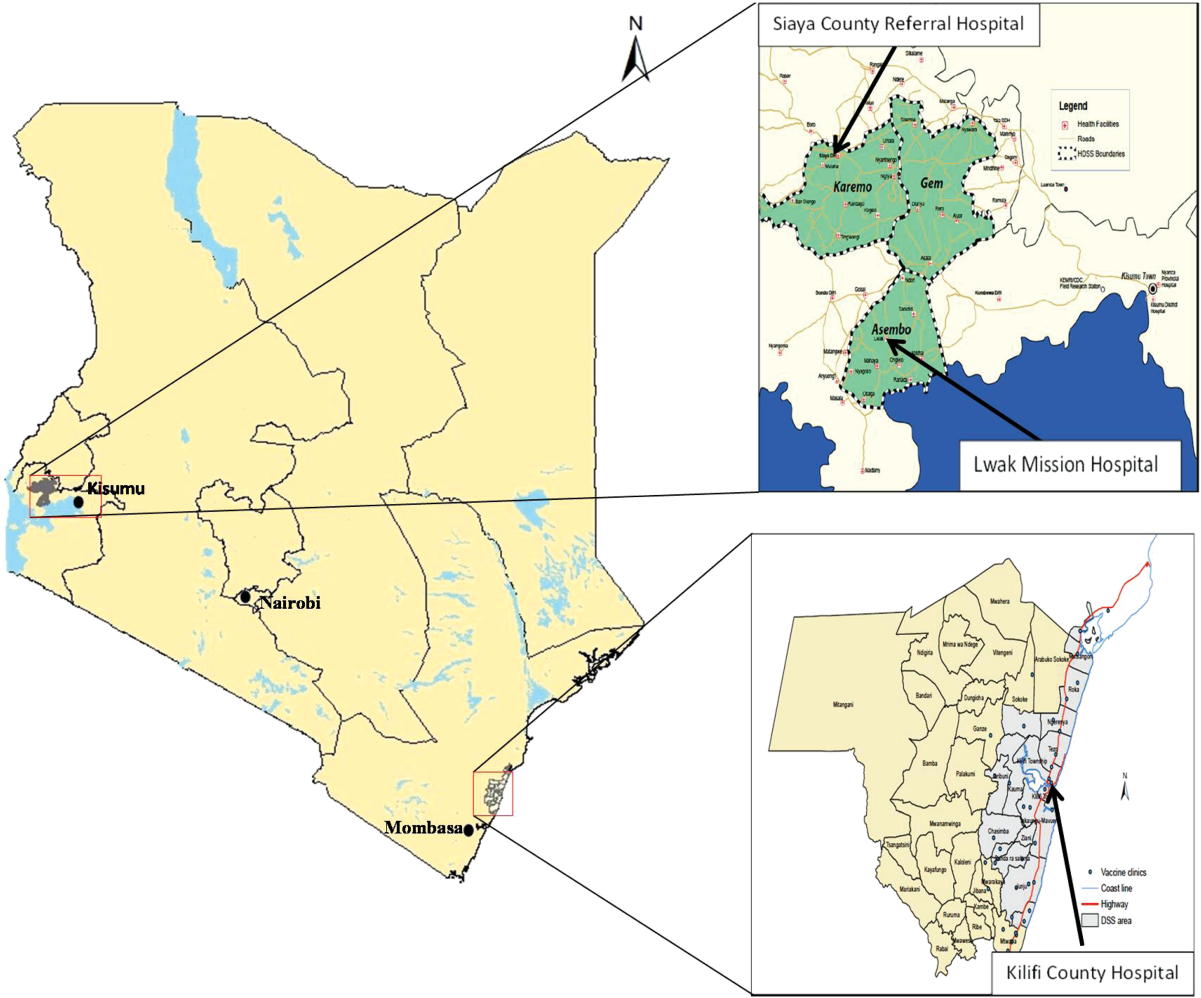
Map showing the Rotavirus Immunization Program Evaluation in Kenya surveillance sites. Abbreviations: DSS, demographic surveillance system; HDSS, health and demographic surveillance system.

Data from 3 surveillance sites were included in the analysis (Figure 1). Kilifi County Hospital (KCH) is located in the coastal region and serves a rural, semirural, and urban population, with a pediatric bed capacity of 40. Rotavirus surveillance at KCH began in 2009 and is implemented by the Kenya Medical Research Institute (KEMRI)–Wellcome Trust Research Programme (KWTRP), a research partnership between KEMRI, the University of Oxford, and the Wellcome Trust, United Kingdom [10, 11]. Siaya County Referral Hospital (SCRH) serves a rural and semirural population with a pediatric bed capacity of 60. Rotavirus surveillance began in 2010, and is carried out by KEMRI’s Centre for Global Health Research (KEMRI-CGHR) in collaboration with the US Centers for Disease Control and Prevention (CDC) as part of a network of WHO rotavirus surveillance sites [12]. Saint Elizabeth Lwak Mission Hospital (LMH) is a private facility with 7 pediatric beds serving a rural population in Asembo, Siaya County. Rotavirus surveillance at LMH is conducted as part of the population-based infectious disease surveillance platform [13].

At all sites, hospitalized children aged 0–59 months were assessed by trained clinical staff; patients with ≥3 loose stools in 24 hours met the AGE case definition. At SCRH, patients with ≥1 episode of unexplained vomiting followed by ≥1 loose stool within 24 hours also met AGE criteria. Cases with onset ≥7 days prior to admission were excluded. Epidemiologic data and a stool sample were collected from AGE case patients. At SCRH and LMH, stool samples were transported on dry ice to the KEMRI-CGHR laboratory located approximately 60 km from the facilities; at KCH samples were immediately transported to the KWTRP laboratory located adjacent to the hospital. Samples at all sites were stored at −80°C before testing.

Vaccination data for AGE case patients enrolled at KCH were primarily obtained through an electronic vaccine registry [14], which captures childhood immunization data in real time from clinics within the area of a health and demographic surveillance system (HDSS) operated by KWTRP [10]; vaccination data are linked to KCH surveillance data using unique HDSS identification numbers. The HDSS operated by KEMRI-CGHR and CDC in Siaya County [15] served as a source of vaccination data for cases enrolled at SCRH and LMH; immunization histories for children <5 years were captured by reviewing child health cards during household data collection rounds occurring 2–3 times per year. For non-HDSS participants enrolled in surveillance at KCH or SCRH, immunization data were captured at the time of enrollment; at LMH, surveillance was restricted to HDSS participants. For HDSS members enrolled in rotavirus surveillance at SCRH or LMH with missing or uncertain vaccination history, household visits were conducted in an attempt to obtain accurate data.

Stool samples from KCH and SCRH were tested at the KWTRP and KEMRI-CGHR laboratories, respectively, using the qualitative enzyme immunoassay ProsPecT (Oxoid Ltd) for detection of rotavirus group A VP6 antigen. For LMH, samples were tested at the KEMRI-CGHR laboratory using either ProSpecT Kit (Oxoid Ltd) or Rotaclone Kit (Meridian Bioscience) for detection of VP6 antigen [16]. Rotavirus-positive stool samples from KCH and SCRH underwent genotyping. KCH samples underwent P and G gene amplification followed by Sanger sequencing and genotype determination of assembled sequences through the online automated tool RotaC on Virus Pathogen Resource [17] at the KWTRP laboratory. Samples from SCRH were sent to the regional WHO rotavirus reference laboratory, Medical Research Council–Diarrhoeal Pathogens Research Unit, South Africa, for genotyping [18]. Samples from LMH were not genotyped.

We used a test-negative case-control study design to evaluate RV1 effectiveness [19, 20], utilizing surveillance data collected from July 2014 through December 2017. To demonstrate a vaccine effectiveness of ≥50% (assuming 80% power at the 5% significance level, vaccine coverage of 70%, and a case to control ratio of 1:2), 105 rotavirus test-positive patients and 210 rotavirus test-negative controls would have been required. Eligibility criteria included: hospitalized with AGE and enrolled in the surveillance platform of a participating site; age-eligible to have received ≥1 RV1 dose prior to illness, with a 14-day window to allow for immunity development (ie, at least 8 weeks of age at enrollment and born at least 6 weeks before vaccine introduction); stool specimen collected and tested for rotavirus; and available vaccination history.

Cases were defined as participants with rotavirus-positive stool, whereas controls had a rotavirus-negative stool. The exposure of interest was RV1 vaccination status (2 vs 0 doses, at least 1 dose vs 0 doses, and exactly 1 dose vs 0 doses). A dose was considered valid if administered >14 days before date of admission. Vaccination status was ascertained using registry/card-confirmed data. However, case patients without a health card whose parents reported no prior receipt of any vaccines were considered to have received zero RV1 doses. Children without vaccination data or missing date of administration were excluded.

Population-level RV1 coverage was calculated using vaccine registry data in Kilifi and card-confirmed vaccination data in the HDSS database in Siaya. To calculate annual coverage, we assessed each child’s age and vaccination status as of December 31 for that year; coverage was defined as the number of children with 2 RV1 doses divided by the total number of children in each age stratum.

Characteristics of cases and controls were compared using χ ^2^ test or Mann-Whitney *U* test. We calculated odds ratios (ORs) for vaccination among cases vs controls using unconditional logistic regression, and vaccine effectiveness as 1 – OR of vaccination × 100%. We a priori adjusted for date of admission, age in weeks, and site as potential confounders. We assessed for additional potential confounders by including variables in the date-, age-, and site-adjusted model; any variable that changed the adjusted odds ratio (aOR) by >10% would be included in the final model. Further analyses included examining (1) duration of protection by measuring effectiveness stratified by age (<12 months and ≥12 months); (2) protection against disease of varying severity using a 20-point clinical Vesikari score, classified as less severe (>11) and severe (≥11) [21]; and (3) effectiveness among children with and without moderate or severe malnutrition. Stunting (low height for age) was used as an indicator of chronic malnutrition, wasting (low weight for height) an indicator of acute malnutrition, and underweight (low weight for age) a composite of acute and chronic malnutrition. All were classified as normal (*z* score ≥ −2.0), moderate (*z* score < −2.0 and ≥ −3.0), or severe (*z* score < −3.0) using WHO growth standards [22, 23]. All models assessing the effectiveness of 2 vs 0 doses were restricted to data from cases and controls who were age-eligible for 2 doses (≥12 weeks of age, since second dose given at age 10 weeks, plus 14-day window for immunity development). Malaria was classified based on the presence or absence of parasites on blood smear. Analyses were carried out using Stata version 13.1 software (StataCorp).

The RIPEK protocol was reviewed and approved by KEMRI’s Scientific and Ethical Review Unit (Scientific Steering Committee number 3049) and the CDC (protocol number 6968). Parents/guardians of participants provided written informed consent for enrollment at each participating platform.

## RESULTS

**Figure 2.**
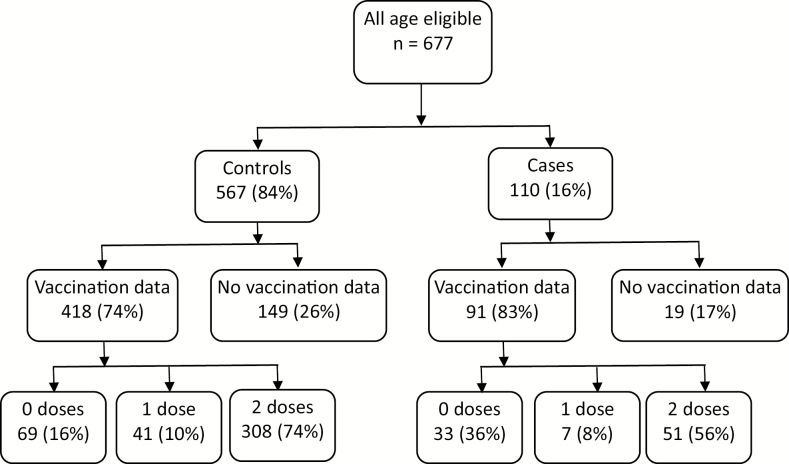
Flowchart for distribution of rotavirus-positive cases and rotavirus-negative controls by vaccination status, among children admitted with diarrhea at 3 hospitals in Kenya, July 2014–December 2017.

From July 2014 to December 2017, 677 children hospitalized with AGE who were age-eligible for vaccination with stool collected and tested were identified from the 3 participating sites. Of these, 110 (16%) were rotavirus-positive cases and 567 (84%) were rotavirus-negative controls (Figure 2). Overall, 509 (75%) had card-confirmed vaccination information (or parental report of nonvaccination), including 91 (83%) cases and 418 (74%) controls. Among 91 rotavirus-positive cases, 33 (36%) were unvaccinated, 7 (8%) had 1 dose, and 51 (56%) were fully vaccinated. Among 418 rotavirus-negative controls, 69 (16%) were unvaccinated, 41 (10%) had 1 dose, and 308 (74%) were fully vaccinated. There were no significant differences between cases and controls in terms of sex, age, site, severity, or nutritional status (Table 1). Cases were less frequently fully vaccinated (56%) than controls (74%).

**Table 1.** Characteristics of Rotavirus-positive Cases and Rotavirus-negative Controls Among Children Admitted With Diarrhea at 3 Hospitals in Kenya, July 2014–December 2017

Variable	Cases (n = 91)	Controls (n = 418)	*P* Value
Male sex	41 (45)	203 (49)	.544
Median age, mo (range)	9.7 (1.4–29.5)	9.8 (1.4–32.0)	.667
Study site			
Kilifi	61 (67)	277 (66)	
Lwak	12 (13)	62 (15)	.916
Siaya	18 (20)	79 (19)	
Month/season of enrollment			
January–March	19 (21)	103 (25)	
April–June	25 (27)	140 (33)	< .001
July–September	39 (43)	93 (22)	
October–December	8 (9)	82 (19)	
Disease severity (Vesikari score)			
Less severe (<11)	57 (63)	279 (67)	.453
Severe (≥11)	34 (37)	139 (33)	
Weight for age^a^			
Normal (*z* ≥ −2)	57 (63)	233 (57)	
Moderate underweight (*z* < −2)	15 (16)	70 (17)	.487
Severe underweight (*z* < −3)	18 (20)	104 (25)	
Height for age^a^			
Normal (*z* ≥ −2)	66 (73)	281 (67)	
Moderate stunting (*z* < −2)	12 (13)	66 (16)	.419
Severe stunting (*z* < −3)	11 (12)	71 (17)	
Weight for height^a^			
Normal (*z* ≥ −2)	63 (70)	245 (58)	
Moderate wasting (*z* < −2)	12 (13)	69 (17)	.189
Severe wasting (*z* < −3)	15 (16)	96 (23)	
Positive malaria blood smear^a^	10 (11)	69 (17)	.212
RV1 dose			
0 doses	33 (36)	69 (15)	
1 dose	7 (8)	41 (10)	<.001
2 doses	51 (56)	308 (74)	

Data are presented as No. (%) unless otherwise indicated. Nutritional status measures of weight for age, height for age, and weight for height were classified as normal (*z* score ≥ −2), moderate (z score < −2 and ≥ −3.0), or severe (*z* score < −3).

Abbreviation: RV1, monovalent rotavirus vaccine.

^a^Missing values excluded from denominator.

**Figure 3.**
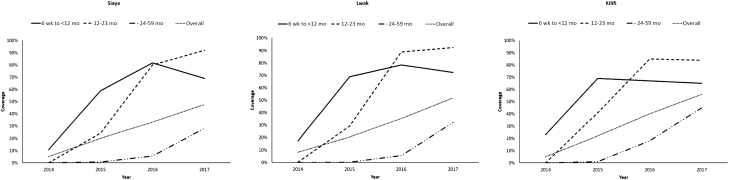
Rotavirus vaccine coverage at different sites by age groups in the populations of 2 health and demographic surveillance system sites between 2014 and 2017.

RV1 coverage (2 doses) increased steadily after introduction in all sites, reaching a high in 2017 of 48% in Siaya, 52% in Lwak, and 56% in Kilifi among children aged 6 weeks to 59 months. Among children aged 12–23 months, coverage was 84%–92% in 2017. Among children aged 6 weeks to <12 months, coverage initially increased but declined in 2017 across all sites (Figure 3).

Among cases, 69 of 91 (75%) had genotype information. The most common G-type was G1 (62%) followed by G2 (28%); the most common P-type was P[8] (67%) followed by P[4] (26%) (Table 2). The most frequent combined genotypes were G1P[8] (61%) and G2P[4] (26%).

**Table 2.** Distribution of Genotypes Among Selected Cases From Inpatient Children Enrolled at 3 Hospitals in Kenya, July 2014–December 2017

	P-Type				
G-Type	P[4]	P[6]	P[8]	P[NT]	Total
G1	0	0	42 (61)	1 (1)	43 (62)
G12	0	0	1 (1)	0	1 (1)
G2	18 (26)	0	1 (1)	0	19 (28)
G3	0	3 (4)	0	0	3 (4)
G3/G9	0	1 (1)	1 (1)	0	2 (3)
GNT	0	0	1 (1)	0	1 (1)
Total	18 (26)	4 (6)	46 (67)	1 (1)	69

Data are presented as No. (%).

Effectiveness of 2 RV1 doses vs zero doses against rotavirus AGE hospitalization was 64% (95% confidence interval [CI], 35%–80%), and for exactly 1 dose it was 54% (95% CI, −20% to 83%) (Table 3). There was no significant difference in the 2-dose effectiveness among children aged <12 months (67% [95% CI, 30%–84%]) and those ≥12 months (72% [95% CI, 10%–91%]). The point estimates for effectiveness against less severe 67% (95%CI, 30%–84%) and more severe 61% (95%CI, 10%–86%) disease were similar; however protection against more severe was not statistically significant.

**Table 3.** Vaccine Effectiveness Estimates^a^ by Different Characteristics for Children at 3 Hospitals in Kenya, July 2014–December 2017

	% Vaccinated					
Characteristic	Cases (n = 91)	Controls (n = 418)	Crude OR (95% CI)	Crude VE, % (95% CI)	Adjusted^b^ OR (95% CI)	Adjusted^b^ VE, % (95% CI)
Among all age-eligible						
2 doses^c^	51/83 (61)	308/365 (84)	0.29 (.17–.50)	**71 (50–83)**	0.36 (.20–.65)	**64 (35–80)**
1 dose^d^	7/40 (18)	41/110 (37)	0.36 (.14–.88)	**59 (12–86)**	0.46 (.17–1.20)	54 (−20 to 83)
≥1 dose	58/91 (64)	349/418 (83)	0.35 (.21–.57)	**65 (43–79)**	0.42 (.24–.73)	**58 (32–78)**
Age^c^						
<12 mo	33/55 (60)	184/218 (84)	0.28 (.14–.53)	**72 (47–86)**	0.33 (.16–.70)	**67 (30–84)**
≥12 mo	18/28 (64)	124/147 (84)	0.33 (.14–.81)	**67 (19–86)**	0.28 (.09–.90)	**72 (10–91)**
Study site^c^						
Kilifi	33/58 (57)	192/237 (81)	0.31 (.18–.57)	**69 (43–82)**	0.37 (.18–.74)	**63 (26–82)**
Siaya	7/14 (50)	58/67 (79)	0.16 (.04–.55)	**84 (45–96)**	0.19 (.04–.79)	**81 (21–96)**
Lwak	11/11 (100)	58/61 (95)	…	…	…	…
Disease severity^c^						
Less severe	34/53 (64)	206/240 (86)	0.30 (.15–.58)	**70 (42–85)**	0.33 (.16–.70)	**67 (30–84)**
Severe	17/30 (57)	102/125 (82)	0.29 (.13–.69)	**71 (31–87)**	0.39 (.14–1.10)	61 (−10 to 86)
Weight for age^c^						
Normal	28/51 (55)	184/210 (87)	0.17 (.09–.34)	**83 (66–91)**	0.16 (.07–.38)	**84 (62–93)**
Moderate underweight	8/14 (57)	55/62 (89)	0.17 (.05–.63)	**83 (37–95)**	0.33 (.08–1.46)	67 (−46 to 92)
Severely underweight	14/17 (82)	67/90 (74)	1.60 (.42–6.08)	−60 (−508 to 58)	1.95 (.46–8.23)	−95 (−723 to 54)
Moderate/severe underweight	22/31 (70)	122/152 (80)	0.64 (.27–1.52)	36 (−52 to 73)	0.90 (.34–2.34)	10 (−134 to 66)
Height for age^c^						
Normal	33/58 (57)	210/247 (85)	0.23 (.12–.44)	**77 (56–88)**	0.25 (.12–.52)	**75 (48–88)**
Moderate stunting	11/12 (92)	46/56 (82)	2.39 (.27–20.70)	−139 (−1970 to 73)	3.97 (.40–39.23)	−297 (−3823 to 60)
Severe stunting	6/11 (55)	52/62 (84)	0.23 (.06–.90)	**77 (10–94)**	0.31 (.07–1.50)	69 (−50 to 93)
Moderate/severe stunting	17/23 (74)	98/118 (83)	0.52 (.19–1.42)	48 (−42 to 81)	0.72 (.24–2.18)	28 (−118 to 76)
Weight for height^c^						
Normal	31/57 (54)	192/218 (88)	0.16 (.08–.31)	**84 (69–92)**	0.16 (.07–.36)	**84 (64–93)**
Moderate wasting	6/11 (55)	53/61 (87)	0.18 (.04–.73)	**82 (27–96)**	0.23 (.04–1.22)	77 (−22 to 96)
Severe wasting	13/14 (93)	59/81 (73)	4.84 (.60–39.27)	−384 (−3827 to 40)	5.59 (.62–50.11)	−459 (−4911 to 38)
Moderate/severe wasting	19/25 (76)	112/142 (79)	0.89 (.33–2.41)	11 (−141 to 67)	1.09 (.37–3.24)	−9 (−224 to 63)
Genotypes^c,e^						
G1P[8]	13/32 (41)	308/365 (84)	0.13 (.06–.27)	**87 (73–94)**	0.40 (.17–.97)	**60 (3–83)**
G2P[4]	15/18 (83)	308/365 (82)	0.93 (.26–3.30)	7 (−230 to 74)	0.71 (.18–2.84)	29 (−184 to 82)

Abbreviations: CI, confidence interval; OR, odds ratio; VE, vaccine effectiveness.

^a^Estimates in bold indicate a VE estimate with a 95% CI with a lower bound >0%.

^b^Adjusted for date of enrollment, age in weeks, and study site.

^c^Model for effectiveness of 2 vs 0 doses. Excludes 7 cases and 41 controls who received exactly 1 dose. Also excludes 1 case and 12 controls aged <12 weeks (therefore not age-eligible for 2 doses).

^d^Model for effectiveness of 1 dose vs 0 doses. Excludes 51 cases and 308 controls who received 2 doses.

^e^Models restricted to cases with listed genotypes.

The effectiveness of 2 RV1 doses among children with normal weight for age was 84% (95% CI, 62%–93%), whereas for moderately or severely underweight children it was 10% (95% CI, −134% to 66%). RV1 effectiveness among those who had normal length/height for age was 75% (95% CI, 48%–88%), whereas no significant protection was observed among those who were moderately or severely stunted (28% [95% CI, −118% to 76%]). Effectiveness among children with normal weight for length/height was 84% (95% CI, 64%–93%), however, for moderately or severely wasted children, no significant protection was observed (−9% [95% CI, −224% to 63%]). The point estimate of effectiveness at the SCRH site (81% [95% CI, 21%–96%]) was higher than that observed in KCH (63% [95% CI, 26%–82%]), although CIs were overlapping. In LMH 100% of cases were vaccinated so the model did not converge. Vaccine effectiveness stratified by genotype showed statistically significant protection against the most common genotype, G1P[8] (60% [95% CI, 3%–83%]).

## DISCUSSION

Using data from ongoing rotavirus surveillance at 3 health facilities located in 2 different regions of Kenya, we demonstrated 64% (95% CI, 35%–80%) effectiveness of 2 doses of RV1 against hospitalization with rotavirus AGE among young children. We found similar estimates of protection among children aged <12 months and ≥12 months. Despite finding robust evidence of effectiveness of the vaccine among well-nourished children, we observed no significant protection for children who were stunted, wasted, or underweight. The lack of effectiveness among malnourished children may help explain the lower efficacy and effectiveness of rotavirus vaccines described in low- and middle-income countries compared to those of high-income settings [5].

The effectiveness against rotavirus hospitalization in this study is similar to that reported from other African countries using RV1, with estimates ranging from 54% to 64% [24–28]. This level of protection is also similar to the range of effectiveness found in African countries using RV5, 35% [29] and 80% [30], although fewer data are available on RV5 in routine immunization programs in Africa. RV1 effectiveness estimates from other African sites have yielded point estimates similar to our results, but without statistically significant CIs [31, 32]. The statistical power of vaccine effectiveness studies can be affected by small numbers and high vaccine coverage [33]. For one of our sites, LMH, the site-specific model for vaccine effectiveness did not converge as 100% of cases were vaccinated. Stool sample collection from potential cases at LMH was suboptimal, particularly in the early phase of this study; cases may have been missed during the immediate postintroduction period, when vaccine coverage was still relatively low (Supplementary Table 1). The discrepancy in effectiveness estimates between sites within this study highlights some of the methodologic challenges of observational vaccine effectiveness studies. Nonetheless, the results using data from all 3 sites provide evidence of robust protection against rotavirus hospitalizations in Kenya.

We found significant RV1 effectiveness with similar point estimates among children aged <12 months and those ≥12 months, providing evidence of protection that persists into the second year of life. The greatest burden of rotavirus infection is experienced in the first year of life, particularly in African settings [34]. Therefore, protection from rotavirus vaccines during the first year of life is critical. However, if protection from vaccine declines over time, the burden of rotavirus disease could shift to an older age group. Rotavirus vaccine clinical trials conducted in Africa raised concerns that protection might wane in the second year of life [4, 35]. Some postintroduction observational studies in African sites have reported a lower effectiveness during the second year of life [28, 32, 36], whereas others have found estimates of protection to be similar among children aged <12 months and ≥12 months [24, 27]; however, several of these studies had limited power to assess age-stratified effectiveness. Continued monitoring of rotavirus disease burden will be important to assess for waning immunity.

Stratifying by nutritional status, we found that among well-nourished children, the vaccine provided significant protection against rotavirus AGE hospitalization; however, among underweight, wasted, and stunted children, there was no significant effectiveness. Studies in Botswana [24] and Malawi [36] have similarly found protection of rotavirus vaccine among well-nourished children (point estimates of 75% and 78%, respectively), but no protection in undernourished children. However, in Malawi it was noted that the effectiveness estimates among well-nourished and stunted children were not statistically significantly different, and in Botswana (as in our study), there was overlap between the CIs for vaccine effectiveness among children with and without malnutrition. Small sample sizes may have limited our ability to fully characterize RV1 protection among malnourished children. Nonetheless, while several factors may be contributing to the lower levels of protection from rotavirus vaccine observed in low-income settings [37], the results of our study and others point to a potential role of nutritional status [38]. Therefore, to optimize efforts to reduce rotavirus- and diarrhea-related morbidity and mortality in Africa, efforts should be made to improve nutrition as well as rotavirus vaccine coverage.

The RV1 is derived from a monovalent G1P[8] strain and has been shown to protect against partially and fully heterotypic genotypes in addition to G1P[8] infections [39, 40]. However, genotype-specific rotavirus vaccine effectiveness data from postintroduction observational studies in Africa are limited. In Malawi, the effectiveness of RV1 was highest for G1P[8] genotypes, and lowest against fully heterotypic strains (although 95% CIs overlapped). Yet in Botswana, the RV1 was found to be significantly protective against G2P[4], which was the predominant genotype. Among genotyped strains in this study, G1P[8] was most common (61%), followed by G2P[4] (26%). We observed statistically significant protection against G1P[8], albeit with wide CIs (60% [95% CI, 3%–83%]) The point estimate of protection against G2P[4] (31%) was lower than that of G1P[8]; however, genotype-specific effectiveness analyses in our study had limited statistical power. Given the potential for RV1 to provide lower levels of protection against non-G1P[8] strains, it is important to monitoring circulating rotavirus strains post–vaccine introduction.

A limitation of our study was the exclusion of 17% of cases and 26% of controls due to lack of card-confirmed vaccination data; excluding children with missing vaccination data can lead to selection bias. However, test-negative designs minimize the potential for selectively collecting vaccination histories based on rotavirus positivity, as vaccination history is gathered before investigators are aware whether an enrolled child will be a case or a control [33]. Our study included only 3 sites, 2 of which are located in the same region; therefore, the findings may not be generalizable to all regions of Kenya. A prolonged period of healthcare worker strikes in 2017 negatively affected enrollment in the surveillance platforms (as well as vaccine coverage). Genotype data were only available for a subset of cases, which limited our ability to examine strain-specific vaccine effectiveness.

This study contributes to the growing body of evidence showing that RV1, when used in routine infant immunization programs in African countries, can effectively prevent severe rotavirus morbidity (hospitalizations) among young children. Although the vaccine effectiveness observed is somewhat lower than that seen in high-income settings, it is consistent with data from other African settings. We did not see evidence of waning protection among children aged ≥12 months. However, our data do suggest that malnutrition may diminish RV1 effectiveness. In areas with a high burden of childhood diarrheal illness and death, a rotavirus vaccine with 60% effectiveness can prevent much illness and save many lives. Efforts to strengthen rotavirus vaccine uptake and improve nutritional status are important to maximize vaccine benefit.

## Supplementary Data

Supplementary materials are available at *Clinical Infectious Diseases* online. Consisting of data provided by the authors to benefit the reader, the posted materials are not copyedited and are the sole responsibility of the authors, so questions or comments should be addressed to the corresponding author.

ciz664_suppl_Supplementary_Table_1

## Notes

***Acknowledgments.*** The authors acknowledge the contribution of the study participants, research teams, and staff members from both the Kilifi and the Kenya Medical Research Institute (KEMRI) Centre for Global Health Research (CGHR) health and demographic surveillance system sites; Ministry of Health personnel at both Kilifi and Siaya county hospitals for their sustained participation and support of research activities; and Britton Tuck for her support of the project. This manuscript has been approved for publication by the director of KEMRI.

***Financial support.*** This study was funded by Gavi, the Vaccine Alliance under the Rotavirus Immunization Programme Evaluation in Kenya study (grant number PP42191214), a collaboration between Emory University, the CDC in Atlanta and in Nairobi, Kenya, the KEMRI–Wellcome Trust Research Programme, the Centre for Geographic Medicine Research-Coast, Kilifi, and KEMRI-CGHR, Kisumu, Kenya. The KEMRI–Wellcome Trust Research Programme (KWTRP) surveillance activities are funded by the Wellcome Trust (grant reference numbers 203077 and 102975). Both the KEMRI-CGHR surveillance at Siaya and the population-based infectious disease surveillance platforms are funded by the CDC while the WHO Regional Office for Africa funded part of the rotavirus surveillance activities.

***Potential conflicts of interest.*** The authors report no potential conflicts of interest. All authors have submitted the ICMJE Form for Disclosure of Potential Conflicts of Interest. Conflicts that the editors consider relevant to the content of the manuscript have been disclosed.
