# Supplementary material for: Effectiveness of Monovalent Rotavirus Vaccine Against Hospitalization With Acute Rotavirus Gastroenteritis in Kenyan Children
Source: Clin Infect Dis. 2019 Jul 20;70(11):2298–305. doi: 10.1093/cid/ciz664 (PMC7245145; doi:10.1093/cid/ciz664)
Supplement: ciz664_suppl_Supplementary_Table_1 [file ciz664_suppl_supplementary_table_1.docx]

Supplementary Table 1. Characteristics of cases and controls by site for in-patients enrolled at 3 hospitals in Kenya July 2014 to December 2017

| **Variable** | | **Kilifi** | | **Lwak** | | **Siaya** | |
| --- | --- | --- | --- | --- | --- | --- | --- |
|  |  | Cases (n=61) | Controls (n=277) | Cases (n=12) | Controls (n=62) | Cases (n=18) | Controls (n=79) |
|  |  | n (%) | n (%) | n (%) | n (%) | n (%) | n (%) |
| Sex | Male | 25 (41) | 118 (42) | 10 (83) | 38(61) | 6 (33) | 47 (59) |
| Median age in months (Range) |  | 9.6 (2.8-29.5) | 9.8 (1.5-32.0) | 12.1 (6.9-19.5) | 12.8 (2.8-31.7) | 8.5 (1.4-17.7) | 8.2 (1.4-20.0) |
| Year of enrollment | 2014 | 0 (0) | 15 (5) | 0 (0) | 0 (0) | 1 (6) | 5 (6) |
|  | 2015 | 37 (61) | 81 (29) | 2 (16) | 9 (14) | 10 (55) | 36 (45) |
|  | 2016 | 20 (32) | 128 (46) | 5 (42) | 21 (34) | 5 (28) | 33 (42) |
|  | 2017 | 4 (7) | 53 (19) | 5 (42) | 32 (52) | 2 (11) | 5 (6) |
| Months/season of enrolment | Jan-Mar | 3 (5) | 68 (25) | 5 (42) | 17 (27) | 11 (61) | 18 (23) |
|  | Apr-Jun | 20 (33) | 89 (32) | 3 (25) | 24 (39) | 2 (11) | 27 (34) |
|  | Jul-Sep | 35 (57) | 62 (22) | 2 (17) | 10 (16) | 2 (11) | 21 (27) |
|  | Oct-Dec | 3 (5) | 58 (21) | 2 (16) | 11 (18) | 3 (17) | 13 (16) |
| Disease severity (Vesikari score) | Less severe | 36 (59) | 169 (61) | 12 (100) | 61 (98) | 9 (50) | 49 (62) |
|  | Severe | 25 (41) | 108 (39) | 0 (0) | 1 (2) | 9 (50) | 30 (38) |
| Stunting* | Normal | 43 (73) | 182 (66) | 8 (66) | 43 (69) | 15 (83) | 56 (71) |
|  | Moderate | 9 (15) | 46 (17) | 2 (17) | 10 (16) | 1 (6) | 10 (13) |
|  | Severe | 7 (12) | 49 (18) | 2 (17) | 9 (15) | 2 (11) | 13 (16) |
| Wasting* | Normal | 41 (67) | 145 (53) | 10 (83) | 48 (79) | 12 (70) | 52 (70) |
|  | Moderate | 9 (15) | 51 (18) | 1 (8) | 7 (11) | 2 (12) | 11 (15) |
|  | Severe | 11 (18) | 79 (29) | 1 (8) | 6 (10) | 3 (18) | 11 (15) |
| Underweight* | Normal | 36 (59) | 143 (52) | 8 (67) | 46 (74) | 13 (76) | 48 (63) |
|  | Moderate | 10 (16) | 44 (16) | 3 (25) | 11 (18) | 2 (12) | 17 (22) |
|  | Severe | 15 (25) | 90 (32) | 1 (8) | 5 (8) | 2 (12) | 11 (15) |
| Malaria* | Positive | 2 (3) | 5 (2) | 2 (17) | 30 (48) | 6 (40) | 34 (46) |
|  | Negative | 59 (98) | 272 (98) | 10 (83) | 32 (52) | 9 (60) | 40 (54) |
| Vaccine dose | 0 doses | 25 (41) | 51 (18) | 0 (0) | 3 (5) | 8 (44) | 15 (19) |
|  | 1 dose | 3 (5) | 34 (12) | 1 (8) | 1 (2) | 3 (17) | 6 (8) |
|  | 2 doses | 33 (54) | 192 (69) | 11 (92) | 58 (93) | 7 (39) | 58 (73) |

*Missing values excluded.
